# Understanding purchasing patterns of alcoholic, alcohol‐free and low‐alcohol drinks: A latent profile analysis

**DOI:** 10.1111/add.70445

**Published:** 2026-04-23

**Authors:** Oscar Rousham, Abigail K. Stevely, John Holmes

**Affiliations:** ^1^ Sheffield Addictions Research Group, School of Medicine and Population Health University of Sheffield Sheffield UK

**Keywords:** alcohol, alcohol‐free, latent profile analysis, low‐alcohol, purchasing patterns, socio‐economic position

## Abstract

**Background and aim:**

Alcohol‐free and low‐alcohol (no/lo) drinks (≤1.2% ABV) are increasingly popular in high‐income countries. Their potential to reduce alcohol‐related harm depends on who buys them, in what quantity and their incorporation into overall drinking patterns. We aimed to (1) compare purchases containing only no/lo drinks, only alcoholic drinks or both, over time between 2018 and 2023; (2) identify subgroups with distinct purchasing patterns in 2023; and (3) describe sociodemographic differences between these subgroups.

**Design:**

Latent profile analysis of cross‐sectional household purchasing data.

**Setting:**

Great Britain, 2018 and 2023.

**Participants:**

Nationally representative samples of 30 401 (2018) and 28 254 (2023) households. 4975 households purchasing no/lo drinks in 2023 were included in the latent profile analysis.

**Measurements:**

Data included off‐trade (i.e. shop) purchasing occasions categorised into no/lo‐only, alcohol‐only or no/lo alongside alcohol. Household characteristics were purchasing frequency, standard servings of no/lo drinks per adult, alcohol risk levels based on weekly units of alcohol purchased per adult (non‐drinker: 0 units; low‐risk: ≤14 units; increasing risk: >14‐ ≤ 35 units; high‐risk: >35 units; 1 unit = 8 g alcohol), age, social class, region and ethnicity.

**Findings:**

From 2018 to 2023, the proportion of purchasing occasions that were alcohol‐only fell from 97% [95% confidence interval (CI) = 97%–97%] to 95% (95% CI = 95%–95%), while no/lo‐only purchases rose from 1.4% (95% CI = 1.3%–1.4%) to 2.7% (95% CI = 2.7%–2.8%) and no/lo alongside alcohol purchases rose from 1.2% (95% CI = 1.2%–1.2%) to 1.9% (95% CI = 1.9%–2.0%). In 2023, no/lo‐only purchases were smaller (median = 6.9 no/lo servings) than no/lo alongside alcohol purchases (median = 6.5 plus 24.5 alcohol units) and alcohol‐only purchases (median = 24.6 units). No/lo‐only purchases occurred earlier in the week, no/lo alongside alcohol purchases peaked on Fridays and Saturdays.

Latent profile analysis identified three classes: *no/lo triers* (53%) averaged 2.1 no/lo servings per adult annually with 95% purchasing no or low‐risk levels of alcohol; *occasional purchasers* (34%) averaged 7.5 servings with 20% purchasing alcohol at increasing or high‐risk levels; *dual purchasers* (13%) averaged 37.8 servings with 39% purchasing alcohol at increasing or high‐risk levels. *Dual purchasers* and *occasional purchasers* were more likely to be older [60% (*P* < 0.001) and 54% (*P* = 0.010) aged ≤55 years, respectively] and white [both 97% (*P* = 0.014 and *P* = 0.0074, respectively)] compared with *no/lo triers* (49% aged ≤55 years, 94% white).

**Conclusions:**

In Great Britain, most households that purchase no/lo drinks appear to do so infrequently and purchase alcohol at low‐risk levels; however, a smaller group of older, higher‐risk households purchase no/lo drinks more frequently.

## INTRODUCTION

Alcohol is a major public health problem, accounting for 5.3% of deaths globally [1], and is the biggest risk factor for death, ill‐health and disability among 15 to 49 year‐olds in England [2]. Alcohol‐related harms are unequally distributed, with lower socio‐economic groups experiencing more harm from less alcohol consumption [3]. Alcohol also imposes a substantial financial burden, costing the National Health Service in England an estimated £4.9bn each year [4]. Although alcohol consumption in the United Kingdom (UK) and other high‐income countries remains high, it has begun to plateau. Per capita intake in the United Kingdom decreased slightly from 11 L in 2010 to 10.7 L in 2020 [5], a trend driven in part by a decline in drinking by younger consumers [6, 7, 8].

In the United Kingdom, alcohol‐free and low‐alcohol (no/lo) drinks, defined as resembling alcoholic beverages in look and taste, but containing 1.2% or less alcohol by volume (≤12 mL ethanol per litre), have seen a rapid increase in popularity since 2018. In 2023, they accounted for 1.3% of sales, up from 0.9% in 2020 [9]. The current UK government has continued the previous government's commitment to supporting industry to increase no/lo drinks availability as a strategy to reduce alcohol consumption and related harms [10, 11]. However, there are concerns that the increased popularity of no/lo drinks may increase alcohol consumption by normalising drinking in new contexts [12] or enabling surrogate marketing, that is, advertising no/lo drinks that share branding with their alcoholic counterpart to circumvent advertising restrictions [13].

To reduce alcohol harms, no/lo drinks must be consumed as a substitute for, rather than in addition to, alcohol and at a scale that meaningfully reduces alcohol consumption [14]. To maximise public health benefits and reduce inequalities, this substitution should occur among heavier drinkers, particularly those of lower socio‐economic position [15]. Evaluating the public health impacts of no/lo drinks, therefore, requires an understanding of how they are incorporated into drinking patterns, who consumes them and in what quantities. As with alcohol, no/lo drinking patterns are likely to vary across intersecting socio‐demographic characteristics [16, 17].

Research on no/lo beverage drinking patterns remains limited. Self‐report data indicate that these drinks are sometimes consumed on their own, for example, when driving or before an early start [14], and that certain socio‐demographic groups are more likely to consume them alongside alcohol [18]. Anecdotal evidence also highlights ‘zebra striping’, occasions, where drinkers alternate between alcoholic and non‐alcoholic drinks to moderate intake [19]. However, no previous studies have used purchasing data to examine how no/lo beverages are integrated into drinking patterns.

There is also emerging evidence that men, heavier drinkers and individuals of higher socio‐economic position (SEP) are more likely to consume or purchase no/lo drinks [18, 20, 21]. Findings regarding age are inconsistent, however, with different studies finding that no/lo consumers are younger [22], older [18] or no evidence for differences in age [21]. These studies are limited by treating no/lo consumption as a binary behaviour (ever versus never consumed) rather than examining the quantity consumed. They also treat no/lo consumers as a homogenous group, overlooking the possibility of distinct subgroups of consumers.

There is a need for a more comprehensive understanding of how no/lo beverages are being incorporated into alcohol consumption patterns and for research that captures the heterogeneity in who consumes no/lo beverages and how much they consume. Given the rapid increase in popularity of no/lo drinks since 2018 there is a need to understand how consumption patterns of no/lo beverages have changed over time.

Households primarily purchase drinks in the off‐trade with the intent to consume them. Therefore, although products may not be consumed straight away (see limitations), this form of purchasing data offers a large‐scale, recall‐bias‐free proxy for consumption patterns. Using household purchasing data for alcoholic and no/lo drinks purchased in the off‐trade (i.e. supermarkets and other stores) we aim to understand the characteristics of no/lo drinks purchasing at two interconnecting levels. At the purchasing occasion level, we compare the characteristics of three purchase types (solely no/lo beverages, solely standard alcoholic beverages and no/lo alongside standard alcoholic beverages) in terms of timing, volume and cost and describe how this has changed between 2018 and 2023 (RQ1). At the household level, we identify no/lo purchasing subgroups based on their annual frequencies of these three purchase types in 2023 (RQ2) and then compare these subgroups in terms of standard alcoholic and no/lo beverage purchasing volumes and socio‐demographic characteristics (RQ3).

## METHODS

The data analysis plan was pre‐registered on the Open Science Framework on 10 February 2025 (https://osf.io/9p7hj/).

### Data

We used data from Kantar's World Panel dataset (KWP) from 2018 and 2023 (the earliest and latest years available to us at the time of analysis). KWP tracks daily off‐trade purchases (i.e. purchases in supermarkets and other stores) for a continuous household purchasing panel of 30 000 nationally representative households in Great Britain. A member of each household scans barcodes of food and drink products brought into the home. We have access to data on alcoholic and no/lo beverage purchases and household socio‐demographics. Households are recruited by stratified quota sampling based on region and socio‐demographics (e.g. household size, children and age of main shopper) and included in the dataset if they meet quality standards including minimum thresholds for recording, purchase volume and spend. Socio‐economic group is part of the weightings used to ensure a representative population, which we use in RQ1. Households that leave the sample are replaced on a continuous basis to maintain a representative sample. Each year is divided into 13 four‐week periods.

For descriptive statistics (RQ1), we included all purchases of alcoholic or no/lo drinks made in 2018 (*n* = 497 821 purchasing occasions by 30 401 households) and 2023 (*n* = 379 205; 28 254). For latent profile analysis (LPA) (RQ2) we looked solely at purchases in 2023 because this period represents the most current data available to us within the fast‐growing no/lo market. We included households present in the dataset for at least three 4‐week periods and with at least one purchasing occasion that included no/lo drinks because our focus was on purchasing patterns that included no/lo beverages. Households without no/lo drinks purchases were reintroduced after model fitting for socio‐demographic comparisons (RQ3).

### Measures

A purchasing occasion was defined as all purchases by a household on a single day and classified into three types: (1) no/lo drinks only; (2) alcohol only; or (3) no/lo drinks alongside alcohol.

At the level of the purchasing occasion, purchase timing was measured as the proportion of daily purchases by number of purchasing occasions by purchase type for each day of the week between 2018 and 2023. Purchase frequency (in 2018 and 2023) was the annual number of occasions across the whole sample for each purchase type. Alcohol volume in units (1 unit = 8 g alcohol), and no/lo volume in servings, defined by drink type [beer: 330 mL; wine: 175 mL; cider: 500 mL; spirits: 50 mL; RTDs (ready to drink beverages): 250 mL] [9], were measured for each purchasing occasion in 2018 and 2023.

At the household level, three behavioural measures were captured: annual purchase frequency by type, alcohol risk category based on the mean units of alcohol purchased per week across the year and number of adults in the household (non‐drinker: 0 units; low‐risk: ≤14 units per adult per week; increasing risk: >14 to ≤35 units; high‐risk: >35 units) and annual no/lo servings per adult.

Household socio‐demographic measures included region (England, Wales and Scotland); social class, recorded by KWP according to the National Readership Survey Classifications [23] and collapsed into higher or intermediate managerial, administrative or professional (AB), junior managerial, administrative or professional (C1), skilled manual workers (C2) and semi‐ or unskilled‐manual workers, casual workers or unemployed (DE); main shopper ethnicity (Asian, Black, Mixed, White and other) and age (<28, 28–34, 35–44, 45–54, 55–64 and 65+ years); and number of adults (calculated from KWP data using household size minus number of children).

## ANALYSIS

### Descriptive statistics (RQ1)

At the purchase level, for each purchase type, we calculated the number of purchases, the proportion of total alcohol or no/lo purchase occasions that this represented and the 95% confidence intervals (CI) for this. Further, we reported the median and interquartile range (IQR) of both cost and volume of alcohol and no/lo drinks across all purchases of that type for 2018 and 2023. For purchase timing, the mean proportion of purchasing occasions that were on each day across all weeks and 95% CI were calculated using Pearson's χ^2^ test. To allow 2018 and 2023 data to be compared like‐for‐like, all descriptive statistics, apart from number of purchasing occasions, were weighted using KWP panel weightings.

### LPA (RQ2–3)

LPA is a clustering method used to split a heterogeneous population into smaller more homogenous groups, called classes, using numerical variables. We used LPA to categorise no/lo‐purchasing households in 2023 according to their purchase frequency across our three purchase types. Models were fit iteratively, starting with one class and adding further classes where model fit improved according to Bayesian information criteria, Akaike information criteria and the bootstrap likelihood ratio test. Models producing latent classes containing less than 5% of participants were excluded, as these typically reflect unsorted cases rather than meaningful subgroups. Model entropy was reported, but not used to choose the final model. All models allowed class‐specific variance (i.e. spread of purchase frequencies about the mean) and covariance (i.e. relationship between the frequency of each of the three purchase types) structures. For subsequent analyses, households were assigned to the class with the highest posterior probability of membership.

We described the household‐level characteristics of each latent class by calculating the mean annual servings of no/lo beverages per adult and the proportion of households in each alcohol risk category. Non‐purchasing households were split into alcohol‐only purchasers and non‐alcohol purchasers and described in the same way. As our aim was descriptive rather than causal, socio‐demographic characteristics of each class were summarised and compared using pairwise statistical tests, treating the largest class as the reference group.

We were unable to weight the LPA analyses because of software constraints. Ethics approval was obtained from the University of Sheffield, application number: 052135. Analyses were conducted in R [24]. *Mplus* [25] via the *Mplusautomation* [26] and *tidyLPA* [27] packages were used to fit latent profile models.

### Missing data

Households were present for a median of 12 of 13 four‐week periods (IQR = 10.25–13). Data exploration suggested missingness was missing at random (MAR), primarily influenced by seasonality, primary shopper's age (younger shoppers had higher missingness) and ethnicity. We used multiple imputation by chained equations (MICE) to estimate missing purchase frequencies and volumes. Imputations were conducted separately for each period to capture seasonal variation and used auxiliary variables reflecting socio‐demographic characteristics (education, social class, region, ethnicity and age) and purchase volume and frequency during observed periods. Volume was imputed after fitting latent profile models, allowing class membership to be included as an auxiliary variable.

Imputation introduces uncertainty in purchase frequency, which affects both the optimal number of latent classes and household classification. To address this, we generated 100 imputed datasets, identified the optimal class number in each using the criteria above and selected the most frequently occurring solution as final. A model with this number of classes was then fit to all 100 datasets, with households assigned according to posterior probability [28].

We also accounted for this uncertainty when calculating latent class characteristics. For household counts, proportions of non‐drinkers and alcohol risk categories, values were calculated within each imputation then averaged. For servings of no/lo drinks, the median for each class was calculated within each imputation then across imputations. Socio‐demographic characteristics were based on the class households were most often assigned to across imputations.

### Changes from the published analysis plan

We made three deviations from the pre‐registered analysis plan (https://osf.io/9p7hj/). First, household‐level purchase frequencies by type were omitted because of space constraints and overlap with RQ2. As a result, the inclusion criteria for RQ1 could be expanded to all purchases in 2018 and 2023. Second, mean annual alcohol purchasing was replaced with alcohol risk levels, which we considered more interpretable. Third, purchase timing was calculated across 2018 to 2023, rather than 2023 alone, to retain useful data.

## RESULTS

### Purchase level characteristics (RQ1)

In 2023 there were 361 708 alcohol‐only purchases, 7562 of no/lo alongside alcohol and 9935 no/lo‐only (Table 1). Therefore, 44% of unweighted no/lo purchases in Great Britain by number of purchasing occasions contained no alcohol, whereas 56% did.

**TABLE 1:** Purchase level characteristics for three purchase types: alcohol only, no/lo drinks alongside alcohol and no/lo drinks only.

Purchase level characteristics	Purchase type					
	Alcohol only		No/lo alongside alcohol		No/lo drinks only	
	2018	2023	2018	2023	2018	2023
No. of purchases [% (95% CI)]	458 349 [97 (97–97)]	361 708 [95 (95–95)]	5948 [1.2 (1.2–1.2)]	7562 [1.9 (1.9–2.0)]	6524 [1.4 (1.3–1.4)]	9935 [2.7 (2.7–2.8)]
Median volume (IQR)						
Alcohol (units of alcohol)	22.3 (36.4)	24.6 (40.6)	21.3 (40.2)	24.5 (43.4)	–	–
No/lo drinks (no. of servings)	–	–	6.3 (8.5)	6.5 (8.3)	7.0 (7.9)	6.9 (9.2)
Median price (£) (IQR)	11.7 (17.8)	14.5 (22)	16.9 (22.5)	23.2 (29.6)	3.94 (4.6)	5.79 (7.56)

Abbreviation: IQR, interquartile range.

From 2018 to 2023, alcohol‐only purchases fell from 97% to 95% of total purchases, no/lo alongside alcohol rose from 1.2% to 1.9% and no/lo‐only saw the largest growth, from 1.4% to 2.7%. Over the same period, median alcohol volume rose from 22.3 to 24.6 units in alcohol‐only purchases and from 21.3 to 24.5 units in no/lo alongside alcohol purchases. Median no/lo drink volumes remained stable, from 6.3 to 6.5 servings when bought alongside alcohol and 7.0 to 6.9 servings when purchased alone. Purchases combining alcohol and no/lo drinks were the largest and most expensive, with alcohol volumes similar to alcohol‐only purchases and no/lo volumes similar to no/lo‐only purchases. In 2023, median prices were £23.20 for mixed purchases, £14.50 for alcohol‐only and £5.79 for no/lo‐only.

**FIGURE 1:**
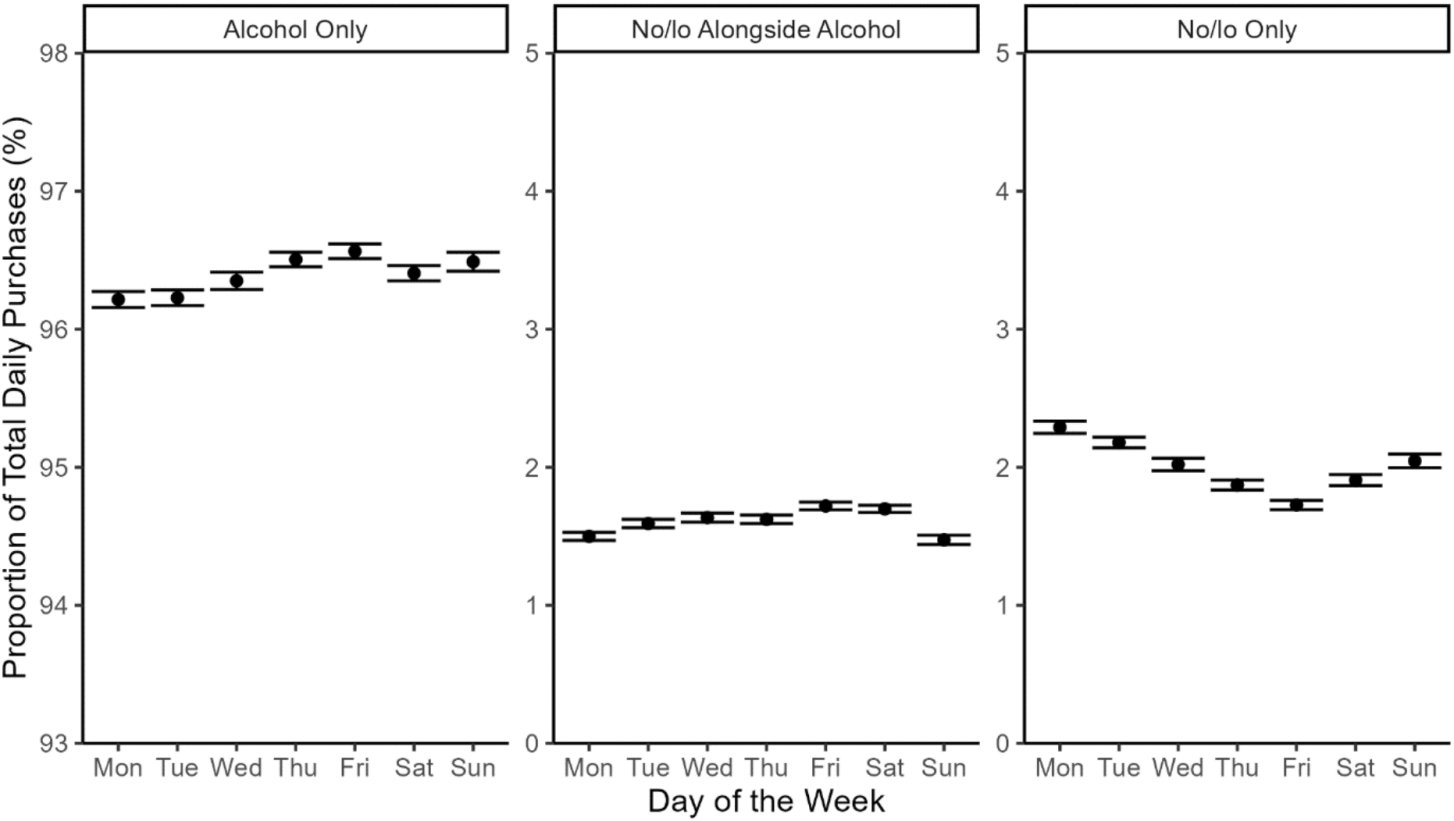
Proportion of total daily purchasing occasions containing alcoholic or no/lo drinks made up by each purchase type by day of the week (%). Points represent the mean proportion; error bars represent the standard error around the mean.

The proportion of purchases by number of purchasing occasions that were alcohol‐only were lowest on Mondays and Tuesdays and higher the rest of the week (Table 2, Figure 1). Purchases of no/lo alongside alcohol peaked on Fridays and Saturdays, whereas no/lo‐only purchases showed the reverse pattern, lowest Thursdays and Fridays and highest Sunday to Wednesday.

**TABLE 2:** The proportion of total purchasing occasions containing alcoholic or no/lo drinks split by day of the week and purchase type.

Day of the week	Proportion of total purchasing occasions containing alcoholic or no/lo drinks, % (95% CI)		
	Alcohol only	No/lo alongside alcohol	No/lo only
Monday	96.3 (96.3–96.4)	1.5 (1.4–1.5)	2.2 (2.1–2.2)
Tuesday	96.3 (96.3–96.4)	1.5 (1.5–1.5)	2.2 (2.1–2.2)
Wednesday	96.5 (96.4–96.5)	1.6 (1.5–1.6)	1.9 (1.9–2.0)
Thursday	96.5 (96.5–96.6)	1.6 (1.6–1.6)	1.9 (1.9–1.9)
Friday	96.6 (96.5–96.6)	1.7 (1.6–1.7)	1.8 (1.7–1.8)
Saturday	96.5 (96.4–96.5)	1.6 (1.6–1.7)	1.9 (1.8–1.9)
Sunday	96.6 (96.6–96.7)	1.4 (1.4–1.4)	2.0 (1.9–2.0)

### No/lo‐purchasing subgroups and their characteristics (RQ2–3)

**FIGURE 2:**
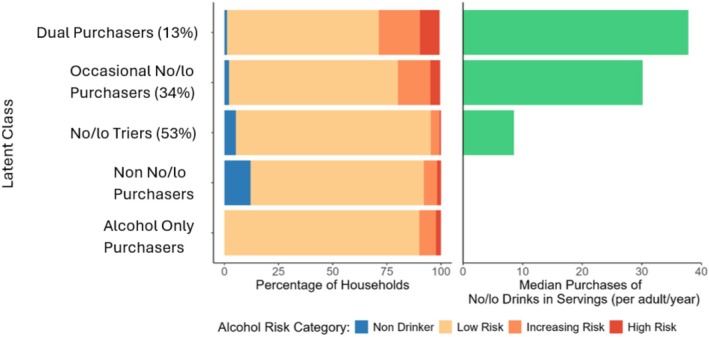
Left hand graph shows the proportion of households in each latent class that purchased alcohol at different risk levels. Risk levels calculated based on each households' alcohol purchases measured in units per adult per week in 2023. Right hand graph shows median purchases of no/lo beverages in servings per adult in 2023 by class.

For households that made at least one no/lo drinks purchase and were present for at least three 4‐week periods in 2023, a three‐class model with variance and covariance differing by class was most often the best fit across imputations (68%) (Table 3). These classes, defined by their household‐level frequency of alcohol‐only, alcohol alongside no/lo and no/lo‐only purchases (Table 4), were: no/lo triers (53% of no/lo households), so called because they averaged just one purchase of no/lo drinks in 2023 comprising 2.1 no/lo servings. This group mostly purchased alcohol at low risk levels (90%) or none (5.2%) (Figure 2). Occasional purchasers (34%) who averaged 7.5 no/lo servings and had higher rates of increasing (16%) and high‐risk (4.4%) alcohol purchasing. Dual purchasers (13%) who were frequent purchasers averaging 37.8 servings and most likely to purchase alcohol at increasing (19%) or high‐risk (10%) levels. Dual purchasers accounted for 58% of all no/lo servings purchased, compared to 28% for occasional purchasers and 14% for no/lo triers.

**TABLE 3:** Model fit criteria from latent profile models iteratively fit with 1, 2, 3 and 4 classes.

Classes	AIC	BIC	Entropy	Class 1 (%)	Class 2 (%)	Class 3 (%)
1	−41 132	−41 073	1.000	1.000	–	–
2	−60 065	−59 941	0.896	25.1	74.9	–
3	−64 332	−64 142	0.842	11.8	53.5	34.7
4	Did not converge					

*Note*: These model fit criteria were randomly selected from models fit using one of our 100 imputed datasets. The model with three classes is the best fitting model according to AIC and BIC. Note that the proportion of households in each class differs slightly for each imputation.

Abbreviations: AIC, Akaike information criteria; BIC, Bayesian information criteria.

**TABLE 4:** Behavioural characteristics by latent class.

Behavioural characteristic	No/lo triers (*n* = 2656 households)	Occasional purchasers (*n* = 1693)	Dual purchasers (*n* = 626)	Alcohol only purchasers (*n* = 18 978)	Non‐no/lo or alcohol purchasers (*n* = 2321)	Total (*n* = 26 272)
Median annual purchase frequency (IQR)						
Alcohol only	9.0 (3.0–20)	22.0 (8.0–44)	22.0 (8.0–47)	8.0 (3.0–21)	–	7 (2.0–20)
No/lo alongside alcohol	0.0 (0.0–0.0)	1 (0.0–2.0)	6.0 (2.0–9.0)	0.0	–	0.0 (0.0–0.0)
No/lo only	1.0 (0.0–1.0)	1.0 (0.0–3.0)	6.0 (2.0–12)	0.0	–	0.0 (0.0–0.0)
Median no/lo servings per adult per year (IQR)	2.1 (1.0–4.3)	7.5 (3.6–14.7)	37.8 (20.3–76.2)	0.0	–	0 (0.0–0.0)
Total volume of no/lo (servings) (%)	9507 (14)	19 877 (28)	40 401 (58)	0	0	69 785 (100)

Abbreviation: IQR, interquartile range.

Regarding socio‐demographic characteristics (Table 5), occasional purchasers were older than no/lo triers (54% versus 49% age 55+ years) and dual purchasers older still (64%). No/lo triers resembled alcohol‐only households in age distribution, but were older than households purchasing neither alcohol nor no/lo.

**TABLE 5:** Socio‐demographic characteristics by latent class.

Socio‐demographic characteristic	No/lo triers (%) (*n* = 2656 households)	Occasional purchasers (%) (*n* = 1693)	*P*‐value	Dual purchasers (%) (*n* = 626)	*P*‐value	Alcohol only purchasers (%) (*n* = 18 978)	*P*‐value	Non‐no/lo or alcohol purchasers (%) (*n* = 2321)	*P*‐value	Total (*n* = 26 272) (%)
Main shopper age			<0.001		0.005		0.01		<0.001	
Under 28 years	0.5	**0.3**		**0.2**		**1.1**		**2.5**		1.2
28–34 years	5.9	**4.0**		**2.1**		**5.7**		**11.2**		6.5
35–44 years	21.3	**18.0**		**14.2**		**19.6**		**26.9**		20.7
45–54 years	23.1	**24.2**		**22.9**		**23.4**		**21.8**		23.1
55–64 years	22.4	**24.1**		**28.3**		**22.6**		**16.5**		21.8
65+ years	26.9	**29.4**		**32.3**		**27.7**		**21.1**		26.7
Ethnicity			0.01		0.007		0.31		<0.001	
Asian	2.8	**1.1**		**0.5**		2.6		**18.9**		5.1
Black	2.0	**1.5**		**1.3**		2.0		**3.9**		2.2
Mixed ethnic background	0.7	**0.5**		**0.9**		0.5		**1.1**		0.6
Other	0.6	**0.4**		**0.2**		0.4		**1.5**		0.6
White	94.0	**96.5**		**97.1**		94.6		**74.6**		91.5
Social class			0.2		0.9		<0.001		<0.001	
AB	23.6	23.9		24.3		**19.0**		**18.3**		19.7
C1	42.6	43.1		45.8		**41.3**		**37.8**		41.1
C2	16.8	15.9		16.6		**17.9**		**15.2**		17.2
DE	17.0	17.1		13.3		**21.8**		**28.6**		22.0
Region			0.6		0.4		0.7		<0.001	
East	11.8	11.9		10.5		11.0		**9.8**		10.9
London	7.4	6.5		6.6		7.6		**13.8**		8.5
Midlands	16.9	19.5		18.4		17.6		**18.4**		17.8
North	26.0	25.0		27.3		26.7		**25.3**		26.3
Scotland	7.8	8.3		9.4		8.1		**8.0**		8.1
South	25.2	24.5		23.6		24.0		**20.3**		23.5
Wales	5.0	4.3		4.2		5.1		**4.4**		4.9

Abbreviations: AB, higher or intermediate managerial, administrative or professional; C1, junior managerial, administrative or professional; C2, skilled manual workers; DE, semi‐ or unskilled‐manual workers, casual workers or unemployed. *P* values are from pairwise statistical tests comparing each group to *no/lo triers*, the largest latent class. Bold indicates significance at the *P* < 0.05 level.

Dual and occasional purchasers both had a significantly higher proportion of White individuals compared to no/lo triers (97% versus 94%). No/lo triers had a similar distribution of ethnicities to alcohol‐only purchasers, but non‐no/lo beverage, non‐alcohol purchasers had a significantly higher proportion of individuals from non‐White backgrounds (24% versus 4%).

No differences in social class were observed between the three no/lo purchasing classes. However, all had a higher proportion from higher social classes than alcohol‐only or non‐alcohol, non‐no/lo purchasers (~24% AB versus 19% and 18%, respectively).

## DISCUSSION

Approximately half (44%) of purchases containing no/lo drinks by number of purchasing occasions in Great Britain were solely no/lo beverages without any alcohol. These purchases tended to be smaller in volume and more often on weekdays than those that contained alcohol. The remaining purchases containing no/lo drinks also contained alcoholic drinks and had the opposite temporal pattern, peaking later in the week on Fridays and Saturdays.

Although off‐trade purchases are not necessarily consumed immediately, the different volume and temporal patterns of each purchase type suggest distinct no/lo drinking occasions. Purchases of solely no/lo drinks earlier in the week could indicate consumption on typically non‐drinking days or full substitution for alcohol. This aligns with findings that people often choose no/lo drinks when they need to wake up early for work or other commitments [14].

The no/lo trier (53%) and occasional purchaser (34%) classes, comprising the majority of no/lo purchasing households, purchased no/lo drinks infrequently, averaging just 2.1 and 7.5 servings per adult per year, respectively. Given this low frequency, even if members of these classes were consuming no/lo drinks as a substitute for alcohol, the scale of this substitution is unlikely to reduce alcohol harms.

This limited potential of no/lo drinks to reduce alcohol‐related harm for most no/lo purchasing households should be weighed against concerns that the increased availability of no/lo drinks may normalise alcohol consumption in new contexts [12] and enable surrogate marketing by the alcohol industry [13]. It should be noted, however, that no/lo drinks are continuing to grow in popularity and patterns and volumes of no/lo purchasing may change as the market grows.

In contrast, the dual purchasers class contained only 13% of no/lo purchasing households yet they accounted for 58% of no/lo purchases by number of servings in 2023. This class also contained the highest proportion of households purchasing alcohol at high‐risk or increasing‐risk levels (29%). This purchasing could, therefore, reduce alcohol harms in a high‐risk group, but only if members of this group are consuming no/lo drinks as a substitute for alcohol.

Existing research on whether people consume no/lo drinks as a substitute for, or in addition to, alcohol is limited. Although free provision [29] and increased availability of no/lo drinks [30] has been shown to reduce alcohol consumption in experimental studies, observational research [31, 32] shows limited evidence for substitution, with small reductions in some subgroups, but not in others. Self‐report data also suggests heterogeneity in this behaviour. A UK survey of past and present no/lo drinkers found that 33% of heavy drinkers consumed no/lo drinks in addition to alcohol, while 25% used them to reduce consumption [14].

The demographics of the three identified classes align with previous research. In line with Perman‐Howe *et al*. [18] and Clarke *et al*. [21], we found no/lo purchasers were more likely to be from higher social classes. Our results suggest that previous mixed findings on the association of age with no/lo purchasing or consumption are not contradictory, but reflect distinct groups of no/lo consumers: We found that one class of no/lo consumers had a similar age profile to non no/lo drinkers, whereas our other two identified classes were older.

Interestingly, we did not identify a class of households that purchased solely no/lo beverages, suggesting that this purchasing behaviour is rare. However, given that our data is at the household level, it is possible that there are individuals who consume solely no/lo drinks but live with alcohol drinkers. This is in accordance with existing research showing that no/lo drinks consumption is more likely in alcohol drinkers than in non‐drinkers [18, 21].

### Strengths and limitations

This study has several strengths. We used purchasing data that is free from recall bias and allows for granular analyses at the level of the purchasing occasion, although households may still under‐report their purchases for other reasons [33]. Applying LPA to these data captured heterogeneity in who purchases no/lo beverages and how much they purchase, overcoming limitations of previous analyses that treat no/lo consumption as a binary outcome by a homogenous group. Multiple imputation enabled the inclusion of a larger sample in the LPA, reducing the risk of bias from missing data. By using many imputations and running latent profile models on each dataset, we also accounted for the uncertainty introduced by the imputation process.

However, there are some limitations to our study. First, data were at the household level and based on purchasing, meaning we cannot determine how the consumption of purchases was distributed across households' members or the characteristics of the households' members who consumed them. Second, our data only covers off‐trade purchases, although the off‐trade accounted for 73% of total alcohol purchases in 2023 [34]. Furthermore, purchasing is not identical to consumption as products might not be consumed immediately, particularly for multi‐packs and large bottles of spirits. However, the dataset's size and longitudinal coverage of both purchasing and non‐purchasing periods helps moderate this.

## CONCLUSION

Although the no/lo market continues to expand, these products currently offer limited population‐level harm reduction, as they are predominantly purchased infrequently and by higher socio‐economic status households who purchase alcohol at low‐risk levels. Future research is needed to determine whether purchasers are consuming no/lo beverages in addition to, or as a substitute for, alcohol. Nevertheless, for the smaller group of older, higher‐risk households found to purchase no/lo drinks more frequently, these beverages could offer a targeted tool for harm reduction. Rather than relying solely on increased no/lo availability to improve public health, policy should focus on promoting and facilitating the substitution of alcohol with no/lo beverages within these higher‐risk groups.

## AUTHOR CONTRIBUTIONS

**Oscar Rousham:** Conceptualization; methodology; formal analysis; visualization; writing—original draft. **Abigail K. Stevely:** Supervision; writing—review and editing; conceptualization. **John Holmes:** Supervision; writing—review and editing; conceptualization.

## DECLARATION OF INTERESTS

O.R. and A.K.S. have no interests to declare. J.H. has received research funding from Alcohol Change UK (ACUK), for an unrelated alcohol research project on young people's use of alcohol‐free and low‐alcohol drinks. ACUK has several commercial partners for its annual Dry January campaign including Walkers Crisps (a PepsiCo brand) and Lucky Saint, an independent brewer of alcohol‐free beers that acquired a pub that sells alcohol in 2023 and became an associate member of the alcohol industry responsibility body The Portman Group in 2025. The partnership with Lucky Saint provides ACUK with less than 0.6% of its overall income.

## Data Availability

This study uses Kantar World Panel market research data which cannot be shared by the authors.
